# 4,4′-(1,8-Naphthalene-1,8-di­yl)dibenzonitrile

**DOI:** 10.1107/S160053681005083X

**Published:** 2010-12-11

**Authors:** Carlos F. Lima, Ligia R. Gomes, Luís M. N. B. F. Santos, John Nicolson Low

**Affiliations:** a Centro de Investigação em Química, Departamento de Química e Bioquímica, Faculdade de Ciências, Universidade do Porto, Rua do Campo Alegre, 687, P-4169 007 Porto, Portugal; b REQUIMTE, Departamento de Química e Bioquímica, Faculdade de Ciências, Universidade do Porto, Rua do Campo Alegre, 687, P-4169 007 Porto, Portugal; c Department of Chemistry, University of Aberdeen, Meston Walk, Old Aberdeen AB24 3UE, Scotland

## Abstract

In the title mol­ecule, C_24_H_14_N_2_, the exterior C—C—C angle of the naphthalene ring system involving the two phenyl-substituted C atoms is 126.06 (11)° and the dihedral angles between the mean plane of the naphthalene ring system and those of the benzene rings are 66.63 (5) and 67.89 (5)°. In the crystal, mol­ecules are linked into a ladders by four weak C—H⋯π inter­actions.

## Related literature

For the structure of the related compound 4-(1-napht­yl)benzonitrile, see: Lima *et al.* (2010 ▶).
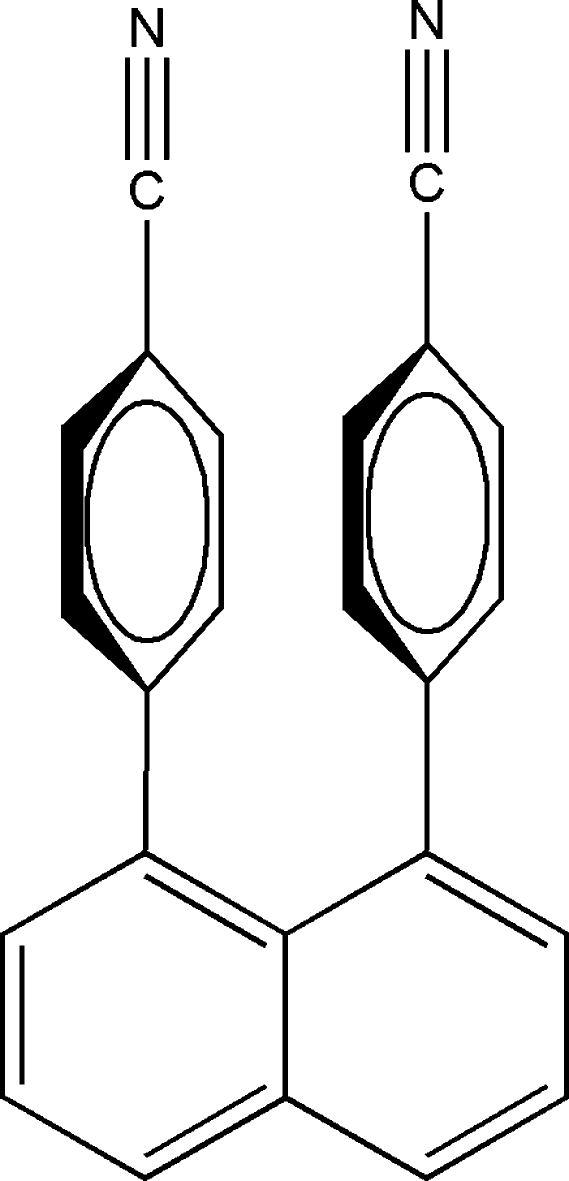


## Experimental

### 

#### Crystal data

C_24_H_14_N_2_
*M* *_r_* = 330.37
Monoclinic, 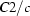

*a* = 17.0872 (9) Å
*b* = 8.2997 (4) Å
*c* = 24.3656 (13) Å
β = 93.795 (2)°
*V* = 3447.9 (3) Å^3^
*Z* = 8
Mo *K*α radiation
μ = 0.08 mm^−1^
*T* = 150 K
0.40 × 0.30 × 0.02 mm

#### Data collection

Bruker SMART APEX diffractometer
Absorption correction: multi-scan (*SADABS*; Bruker, 2004 ▶) *T*_min_ = 0.971, *T*_max_ = 0.999
11422 measured reflections
4634 independent reflections
3482 reflections with *I* > 2σ(*I*)
*R*_int_ = 0.031

#### Refinement

*R*[*F* ^2^ > 2σ(*F* ^2^)] = 0.048
*wR*(*F* ^2^) = 0.131
*S* = 1.04
4634 reflections
235 parameters
H-atom parameters constrained
Δρ_max_ = 0.38 e Å^−3^
Δρ_min_ = −0.26 e Å^−3^

### 

Data collection: *APEX2* (Bruker, 2004 ▶); cell refinement: *APEX2* and *SAINT* (Bruker, 2004 ▶); data reduction: *SAINT*; program(s) used to solve structure: *SHELXS97* (Sheldrick, 2008 ▶); program(s) used to refine structure: *SHELXL97* (Sheldrick, 2008 ▶) and *OSCAIL* (McArdle *et al.*, 2004 ▶); molecular graphics: *PLATON* (Spek, 2009 ▶); software used to prepare material for publication: *SHELXL97*.

## Supplementary Material

Crystal structure: contains datablocks global, I. DOI: 10.1107/S160053681005083X/lh5183sup1.cif

Structure factors: contains datablocks I. DOI: 10.1107/S160053681005083X/lh5183Isup2.hkl

Additional supplementary materials:  crystallographic information; 3D view; checkCIF report

## Figures and Tables

**Table 1:** Hydrogen-bond geometry (Å, °) *Cg*1 and *Cg*2 are the centroids of the C1–C10 and C8–C10 rings, respectively.

*D*—H⋯*A*	*D*—H	H⋯*A*	*D*⋯*A*	*D*—H⋯*A*
C12—H12⋯*Cg*2^i^	0.95	2.75	3.6147 (13)	152
C16—H16⋯*Cg*2^ii^	0.95	2.92	3.6539 (15)	135
C82—H82⋯*Cg*1^i^	0.95	2.83	3.6180 (15)	141
C86—H86⋯*Cg*1^ii^	0.95	2.83	3.6614 (13)	147

Symmetry codes: (i) 
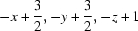
; (ii) 
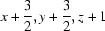
.

**Fig. 1.**
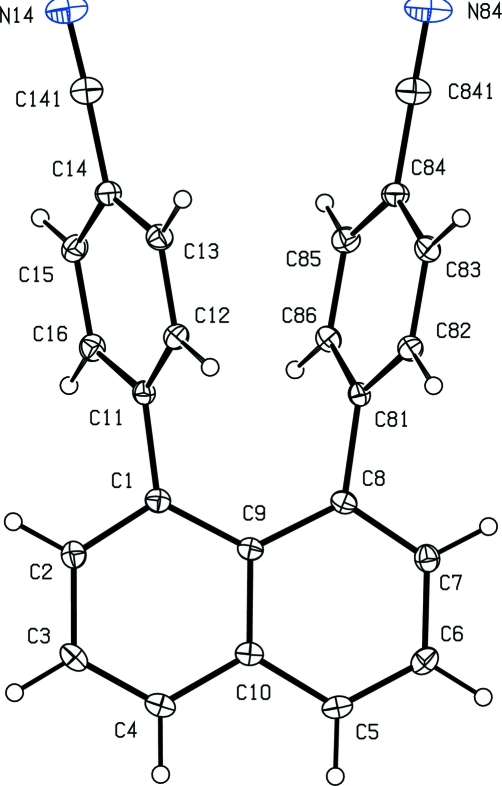
The molecular structure of the title compound with our numbering scheme. Displacement ellipsoids are drawn at the 30% probability level.

**Fig. 2.**
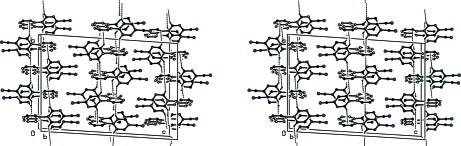
Stereoview of the ladders formed by C—H···π interactions (dashed lines). Hydrogen atoms not involved in the motifs are not included.

## supplementary crystallographic information

### Comment

The exterior C1-C9-C8 angle of the naphthalene ring in C_24_H_14_N_2_ is significantly larger, 126.106 (11)°, than that found in the two independent molecules of the single phenyl substituted compound, 4-(1-naphtyl)benzonitrile (Lima *et al.*, 2010), with values of  123.17 (11)° and 123.21 (10)° as are the angles C9—C1—C11 and C9—C8—C81, 124.93 (10)° and 124.79 (11)° as compared to the values for the single phenyl susbstituent of 121.463 (11)° and 121.47 (10)°.

The dihedral angles between the mean planes of the naphthalene ring and the C11—C16 ring and the C81—C86 rings are 66.33 (5)° and 67.89 (5)° respectively. These angles are significantly larger than those found for the single phenyl substituent in the two molecules of 4-(1-naphtyl)benzonitrile in which the naphthalene rings form dihedral angles of 60.28 (3)° and 60.79 (3)° for molecules 1 and 2 respectively.

C12 and C82 are linked *via* C—H···.π interactions to the centres-of-gravity of the rings C8—C10 and C1—C10 at (3/2 - *x*,3/2 - *y*,1 - *y*) respectively and C16 and C86 are linked *via* C—H···π interactions to the centres-of-gravity of the rings C8—C10 and C1—C10 at (1 - *x*,1 - *y*,1 - *y*) respectively, Table 1. The molecules are thus linked into ladders with the molecules being stacked alternately head-to-tail as the rungs with the cyano groups and atoms C4 and C5 of the naphthalene groups pointing outwards. Alternate ladders run parallel to (110) and (-110). There is an solvent accessible void of 47 Å^3^ in the structure lying between the ladders. These contains no residual electron density. There is no π···π stacking nor are there C—H···N hydrogen bonds.

### Refinement

H atoms were treated as riding atoms with C—H(aromatic), 0.95 Å, with *U*_iso_ = 1.2Ueq(C). The positions of the H atoms were calculated and checked on a difference map during the refinement.

### Crystal data

**Table:**

C_24_H_14_N_2_	*F*(000) = 1376
*M**_r_* = 330.37	*D*_x_ = 1.273 Mg m^−^^3^
Monoclinic, *C*2/*c*	Mo *K*α radiation, λ = 0.71073 Å
Hall symbol: -C 2yc	Cell parameters from 205 reflections
*a* = 17.0872 (9) Å	θ = 7.4–29.2°
*b* = 8.2997 (4) Å	µ = 0.08 mm^−^^1^
*c* = 24.3656 (13) Å	*T* = 150 K
β = 93.795 (2)°	Plate, white
*V* = 3447.9 (3) Å^3^	0.40 × 0.30 × 0.02 mm
*Z* = 8	

### Data collection

**Table:**

Bruker SMART APEX diffractometer	4634 independent reflections
Radiation source: fine-focus sealed tube	3482 reflections with *I* > 2σ(*I*)
graphite	*R*_int_ = 0.031
Detector resolution: 8.33 pixels mm^-1^	θ_max_ = 29.2°, θ_min_ = 3.0°
ω scans	*h* = −22→23
Absorption correction: multi-scan (*SADABS*; Bruker, 2004)	*k* = −11→6
*T*_min_ = 0.971, *T*_max_ = 0.999	*l* = −24→33
11422 measured reflections	

### Refinement

**Table:**

Refinement on *F*^2^	Primary atom site location: structure-invariant direct methods
Least-squares matrix: full	Secondary atom site location: difference Fourier map
*R*[*F*^2^ > 2σ(*F*^2^)] = 0.048	Hydrogen site location: inferred from neighbouring sites
*wR*(*F*^2^) = 0.131	H-atom parameters constrained
*S* = 1.04	*w* = 1/[σ^2^(*F*_o_^2^) + (0.0635*P*)^2^ + 1.3141*P*] where *P* = (*F*_o_^2^ + 2*F*_c_^2^)/3
4634 reflections	(Δ/σ)_max_ = 0.001
235 parameters	Δρ_max_ = 0.38 e Å^−^^3^
0 restraints	Δρ_min_ = −0.26 e Å^−^^3^

### Special details

**Table:**

Geometry. All e.s.d.'s (except the e.s.d. in the dihedral angle between two l.s. planes) are estimated using the full covariance matrix. The cell e.s.d.'s are taken into account individually in the estimation of e.s.d.'s in distances, angles and torsion angles; correlations between e.s.d.'s in cell parameters are only used when they are defined by crystal symmetry. An approximate (isotropic) treatment of cell e.s.d.'s is used for estimating e.s.d.'s involving l.s. planes.
Refinement. Refinement of *F*^2^ against ALL reflections. The weighted *R*-factor *wR* and goodness of fit *S* are based on *F*^2^, conventional *R*-factors *R* are based on *F*, with *F* set to zero for negative *F*^2^. The threshold expression of *F*^2^ > σ(*F*^2^) is used only for calculating *R*-factors(gt) *etc*. and is not relevant to the choice of reflections for refinement. *R*-factors based on *F*^2^ are statistically about twice as large as those based on *F*, and *R*- factors based on ALL data will be even larger.

### Fractional atomic coordinates and isotropic or equivalent isotropic displacement parameters (Å^2^)

**Table:**

	*x*	*y*	*z*	*U*_iso_*/*U*_eq_	
N14	0.69235 (9)	0.27151 (19)	0.76246 (5)	0.0427 (4)	
N84	0.60515 (8)	0.8449 (2)	0.77878 (5)	0.0449 (4)	
C1	0.64595 (7)	0.48335 (15)	0.49000 (5)	0.0182 (3)	
C2	0.66041 (7)	0.35609 (16)	0.45575 (5)	0.0216 (3)	
H2	0.6753	0.2553	0.4717	0.026*	
C3	0.65391 (8)	0.36994 (17)	0.39800 (5)	0.0237 (3)	
H3	0.6644	0.2800	0.3755	0.028*	
C4	0.63235 (8)	0.51393 (17)	0.37489 (5)	0.0228 (3)	
H4	0.6277	0.5238	0.3360	0.027*	
C5	0.59492 (7)	0.79704 (17)	0.38171 (5)	0.0225 (3)	
H5	0.5909	0.8029	0.3427	0.027*	
C6	0.57980 (8)	0.93033 (17)	0.41173 (5)	0.0238 (3)	
H6	0.5655	1.0288	0.3939	0.029*	
C7	0.58549 (7)	0.92080 (16)	0.46957 (5)	0.0219 (3)	
H7	0.5755	1.0148	0.4902	0.026*	
C8	0.60507 (7)	0.77995 (15)	0.49740 (5)	0.0183 (3)	
C9	0.62271 (7)	0.63717 (15)	0.46689 (5)	0.0173 (3)	
C10	0.61666 (7)	0.64945 (16)	0.40782 (5)	0.0192 (3)	
C11	0.65558 (7)	0.44683 (15)	0.55012 (5)	0.0182 (3)	
C12	0.71645 (7)	0.51380 (15)	0.58382 (5)	0.0199 (3)	
H12	0.7517	0.5876	0.5687	0.024*	
C13	0.72607 (7)	0.47384 (16)	0.63911 (5)	0.0220 (3)	
H13	0.7670	0.5212	0.6620	0.026*	
C14	0.67492 (8)	0.36320 (16)	0.66076 (5)	0.0226 (3)	
C15	0.61429 (8)	0.29422 (16)	0.62760 (6)	0.0235 (3)	
H15	0.5795	0.2194	0.6427	0.028*	
C16	0.60519 (8)	0.33568 (16)	0.57243 (5)	0.0222 (3)	
H16	0.5643	0.2880	0.5496	0.027*	
C81	0.60545 (7)	0.78930 (15)	0.55880 (5)	0.0180 (3)	
C82	0.66047 (8)	0.88695 (16)	0.58767 (5)	0.0213 (3)	
H82	0.6978	0.9448	0.5682	0.026*	
C83	0.66135 (8)	0.90069 (16)	0.64442 (5)	0.0227 (3)	
H83	0.6997	0.9656	0.6638	0.027*	
C84	0.60552 (8)	0.81858 (17)	0.67278 (5)	0.0227 (3)	
C85	0.54901 (7)	0.72304 (17)	0.64435 (5)	0.0228 (3)	
H85	0.5106	0.6680	0.6637	0.027*	
C86	0.54928 (7)	0.70913 (16)	0.58781 (5)	0.0201 (3)	
H86	0.5108	0.6443	0.5685	0.024*	
C141	0.68499 (8)	0.31483 (19)	0.71759 (6)	0.0287 (3)	
C841	0.60570 (8)	0.83269 (19)	0.73182 (6)	0.0297 (3)	

### Atomic displacement parameters (Å^2^)

**Table:**

	*U*^11^	*U*^22^	*U*^33^	*U*^12^	*U*^13^	*U*^23^
N14	0.0462 (8)	0.0557 (10)	0.0262 (7)	−0.0037 (7)	0.0020 (6)	0.0074 (6)
N84	0.0411 (8)	0.0708 (11)	0.0227 (7)	−0.0121 (7)	0.0020 (6)	0.0000 (7)
C1	0.0156 (5)	0.0202 (6)	0.0184 (6)	−0.0023 (5)	−0.0006 (4)	−0.0009 (5)
C2	0.0210 (6)	0.0199 (7)	0.0239 (7)	0.0000 (5)	0.0007 (5)	−0.0011 (5)
C3	0.0229 (6)	0.0258 (7)	0.0227 (7)	−0.0014 (5)	0.0042 (5)	−0.0083 (5)
C4	0.0214 (6)	0.0298 (7)	0.0173 (6)	−0.0028 (5)	0.0018 (5)	−0.0033 (5)
C5	0.0210 (6)	0.0294 (7)	0.0169 (6)	−0.0014 (5)	0.0001 (5)	0.0039 (5)
C6	0.0216 (6)	0.0244 (7)	0.0253 (7)	0.0025 (5)	0.0020 (5)	0.0061 (5)
C7	0.0202 (6)	0.0210 (7)	0.0247 (7)	0.0007 (5)	0.0036 (5)	−0.0012 (5)
C8	0.0157 (5)	0.0221 (7)	0.0171 (6)	−0.0008 (5)	0.0023 (4)	−0.0016 (5)
C9	0.0142 (5)	0.0215 (6)	0.0161 (6)	−0.0009 (4)	0.0004 (4)	−0.0008 (5)
C10	0.0155 (5)	0.0248 (7)	0.0173 (6)	−0.0027 (5)	0.0013 (4)	−0.0001 (5)
C11	0.0188 (6)	0.0175 (6)	0.0182 (6)	0.0033 (5)	0.0014 (4)	−0.0010 (5)
C12	0.0182 (6)	0.0204 (6)	0.0209 (6)	0.0007 (5)	0.0006 (5)	0.0011 (5)
C13	0.0202 (6)	0.0240 (7)	0.0211 (6)	0.0016 (5)	−0.0025 (5)	−0.0017 (5)
C14	0.0256 (6)	0.0235 (7)	0.0188 (6)	0.0043 (5)	0.0021 (5)	0.0009 (5)
C15	0.0244 (6)	0.0213 (7)	0.0249 (7)	−0.0006 (5)	0.0033 (5)	0.0032 (5)
C16	0.0213 (6)	0.0207 (7)	0.0241 (7)	−0.0026 (5)	−0.0006 (5)	−0.0007 (5)
C81	0.0193 (6)	0.0170 (6)	0.0179 (6)	0.0034 (5)	0.0023 (4)	−0.0013 (5)
C82	0.0235 (6)	0.0202 (6)	0.0206 (6)	−0.0011 (5)	0.0033 (5)	0.0001 (5)
C83	0.0247 (6)	0.0213 (7)	0.0217 (6)	−0.0016 (5)	−0.0009 (5)	−0.0022 (5)
C84	0.0250 (6)	0.0253 (7)	0.0179 (6)	0.0026 (5)	0.0022 (5)	0.0008 (5)
C85	0.0202 (6)	0.0264 (7)	0.0222 (6)	0.0006 (5)	0.0041 (5)	0.0017 (5)
C86	0.0179 (6)	0.0214 (7)	0.0212 (6)	−0.0001 (5)	0.0020 (5)	−0.0025 (5)
C141	0.0290 (7)	0.0336 (8)	0.0235 (7)	−0.0007 (6)	0.0019 (5)	0.0020 (6)
C841	0.0277 (7)	0.0384 (9)	0.0229 (7)	−0.0045 (6)	0.0011 (5)	0.0005 (6)

### Geometric parameters (Å, °)

**Table:**

N14—C141	1.1499 (19)	C11—C12	1.3971 (17)
N84—C841	1.1496 (19)	C12—C13	1.3867 (17)
C1—C2	1.3785 (18)	C12—H12	0.9500
C1—C9	1.4405 (17)	C13—C14	1.3951 (19)
C1—C11	1.4943 (17)	C13—H13	0.9500
C2—C3	1.4088 (18)	C14—C15	1.3938 (18)
C2—H2	0.9500	C14—C141	1.4411 (18)
C3—C4	1.3614 (19)	C15—C16	1.3864 (18)
C3—H3	0.9500	C15—H15	0.9500
C4—C10	1.4176 (18)	C16—H16	0.9500
C4—H4	0.9500	C81—C82	1.3957 (17)
C5—C6	1.3603 (19)	C81—C86	1.3976 (18)
C5—C10	1.4185 (18)	C82—C83	1.3864 (18)
C5—H5	0.9500	C82—H82	0.9500
C6—C7	1.4085 (18)	C83—C84	1.3926 (19)
C6—H6	0.9500	C83—H83	0.9500
C7—C8	1.3814 (18)	C84—C85	1.3973 (18)
C7—H7	0.9500	C84—C841	1.4431 (18)
C8—C9	1.4413 (17)	C85—C86	1.3829 (18)
C8—C81	1.4977 (17)	C85—H85	0.9500
C9—C10	1.4396 (17)	C86—H86	0.9500
C11—C16	1.3965 (18)		
			
C2—C1—C9	119.88 (11)	C13—C12—H12	119.6
C2—C1—C11	115.18 (11)	C11—C12—H12	119.6
C9—C1—C11	124.93 (11)	C12—C13—C14	119.19 (12)
C1—C2—C3	122.46 (12)	C12—C13—H13	120.4
C1—C2—H2	118.8	C14—C13—H13	120.4
C3—C2—H2	118.8	C15—C14—C13	120.76 (12)
C4—C3—C2	119.08 (12)	C15—C14—C141	118.65 (13)
C4—C3—H3	120.5	C13—C14—C141	120.58 (12)
C2—C3—H3	120.5	C16—C15—C14	119.44 (12)
C3—C4—C10	121.23 (12)	C16—C15—H15	120.3
C3—C4—H4	119.4	C14—C15—H15	120.3
C10—C4—H4	119.4	C15—C16—C11	120.60 (12)
C6—C5—C10	120.96 (12)	C15—C16—H16	119.7
C6—C5—H5	119.5	C11—C16—H16	119.7
C10—C5—H5	119.5	C82—C81—C86	118.93 (11)
C5—C6—C7	119.30 (12)	C82—C81—C8	119.46 (11)
C5—C6—H6	120.4	C86—C81—C8	121.52 (11)
C7—C6—H6	120.4	C83—C82—C81	120.86 (12)
C8—C7—C6	122.49 (12)	C83—C82—H82	119.6
C8—C7—H7	118.8	C81—C82—H82	119.6
C6—C7—H7	118.8	C82—C83—C84	119.47 (12)
C7—C8—C9	119.65 (11)	C82—C83—H83	120.3
C7—C8—C81	115.56 (11)	C84—C83—H83	120.3
C9—C8—C81	124.79 (11)	C83—C84—C85	120.37 (12)
C10—C9—C1	116.95 (11)	C83—C84—C841	119.90 (12)
C10—C9—C8	116.99 (11)	C85—C84—C841	119.73 (12)
C1—C9—C8	126.06 (11)	C86—C85—C84	119.56 (12)
C4—C10—C5	119.01 (12)	C86—C85—H85	120.2
C4—C10—C9	120.40 (12)	C84—C85—H85	120.2
C5—C10—C9	120.59 (12)	C85—C86—C81	120.79 (12)
C16—C11—C12	119.22 (12)	C85—C86—H86	119.6
C16—C11—C1	119.01 (11)	C81—C86—H86	119.6
C12—C11—C1	121.67 (11)	N14—C141—C14	177.90 (17)
C13—C12—C11	120.78 (12)	N84—C841—C84	179.27 (18)
			
C9—C1—C2—C3	0.44 (19)	C2—C1—C11—C12	111.39 (14)
C11—C1—C2—C3	179.92 (11)	C9—C1—C11—C12	−69.16 (16)
C1—C2—C3—C4	−0.18 (19)	C16—C11—C12—C13	−1.45 (19)
C2—C3—C4—C10	0.18 (19)	C1—C11—C12—C13	−177.65 (12)
C10—C5—C6—C7	−0.33 (19)	C11—C12—C13—C14	1.13 (19)
C5—C6—C7—C8	−0.80 (19)	C12—C13—C14—C15	−0.6 (2)
C6—C7—C8—C9	1.79 (19)	C12—C13—C14—C141	177.82 (13)
C6—C7—C8—C81	−177.71 (11)	C13—C14—C15—C16	0.4 (2)
C2—C1—C9—C10	−0.67 (17)	C141—C14—C15—C16	−178.07 (13)
C11—C1—C9—C10	179.91 (11)	C14—C15—C16—C11	−0.7 (2)
C2—C1—C9—C8	179.44 (12)	C12—C11—C16—C15	1.22 (19)
C11—C1—C9—C8	0.02 (19)	C1—C11—C16—C15	177.52 (12)
C7—C8—C9—C10	−1.62 (17)	C7—C8—C81—C82	−65.72 (15)
C81—C8—C9—C10	177.84 (11)	C9—C8—C81—C82	114.80 (14)
C7—C8—C9—C1	178.27 (11)	C7—C8—C81—C86	110.83 (14)
C81—C8—C9—C1	−2.27 (19)	C9—C8—C81—C86	−68.65 (16)
C3—C4—C10—C5	179.57 (12)	C86—C81—C82—C83	2.07 (19)
C3—C4—C10—C9	−0.45 (19)	C8—C81—C82—C83	178.71 (12)
C6—C5—C10—C4	−179.61 (12)	C81—C82—C83—C84	−1.37 (19)
C6—C5—C10—C9	0.41 (19)	C82—C83—C84—C85	0.0 (2)
C1—C9—C10—C4	0.68 (16)	C82—C83—C84—C841	−179.70 (13)
C8—C9—C10—C4	−179.42 (11)	C83—C84—C85—C86	0.64 (19)
C1—C9—C10—C5	−179.34 (11)	C841—C84—C85—C86	−179.66 (12)
C8—C9—C10—C5	0.56 (17)	C84—C85—C86—C81	0.08 (19)
C2—C1—C11—C16	−64.81 (15)	C82—C81—C86—C85	−1.42 (18)
C9—C1—C11—C16	114.64 (14)	C8—C81—C86—C85	−177.98 (11)

### Hydrogen-bond geometry (Å, °)

**Table:**

Cg1and Cg2 are the centroids of the C1–C10 and C8–C10 rings, respectively.

**Table:**

*D*—H···*A*	*D*—H	H···*A*	*D*···*A*	*D*—H···*A*
C12—H12···Cg2^i^	0.95	2.75	3.6147 (13)	152
C16—H16···Cg2^ii^	0.95	2.92	3.6539 (15)	135
C82—H82···Cg1^i^	0.95	2.83	3.6180 (15)	141
C86—H86···Cg1^ii^	0.95	2.83	3.6614 (13)	147

Symmetry codes: (i) −*x*+3/2, −*y*+3/2, −*z*+1; (ii) *x*+3/2, *y*+3/2, *z*+1.

## References

[bb1] Bruker (2004). *APEX2*, *SAINT* and *SADABS* Bruker AXS Inc., Madison, Wisconsin, USA.

[bb2] Lima, C. F., Gomes, L. R., Santos, L. M. N. B. F. & Low, J. N. (2010). *Acta Cryst.* E**66**, o3289. 10.1107/S1600536810042108 PMC3011424 21589568

[bb3] McArdle, P., Gilligan, K., Cunningham, D., Dark, R. & Mahon, M. (2004). *CrystEngComm*, **6**, 303–309.

[bb4] Sheldrick, G. M. (2008). *Acta Cryst.* A**64**, 112–122. 10.1107/S0108767307043930 18156677

[bb5] Spek, A. L. (2009). *Acta Cryst.* D**65**, 148–155. 10.1107/S090744490804362X PMC2631630 19171970

